# Supplementary material for: Efficient and automatic methods for flexible regression on spatiotemporal data, with applications to groundwater monitoring
Source: Environmetrics. 2015 Jun 18;26(6):431–41. doi: 10.1002/env.2347 (PMC4744788; doi:10.1002/env.2347)
Supplement: Supplementary file 1 — Supporting Info item [file ENV-26-431-s001.pdf]

# Efficient and automatic methods for flexible regression on spatiotemporal data, with applications to groundwater monitoring

## Supplementary information

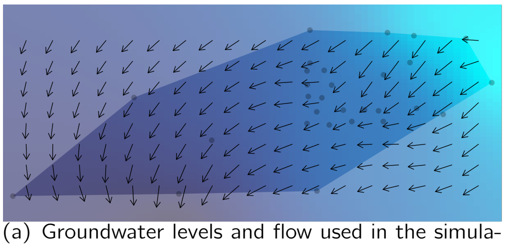

(a) Groundwater levels and flow used in the simulation

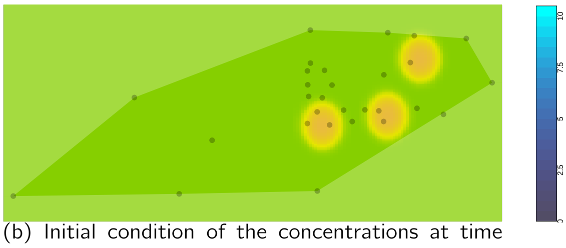

(b) Initial condition of the concentrations at time $t = 0$

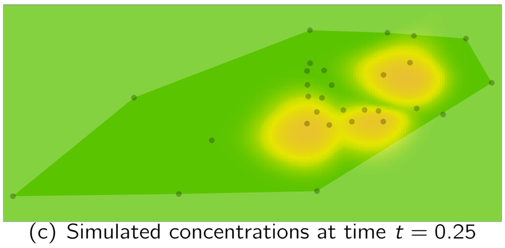

(c) Simulated concentrations at time $t = 0.25$

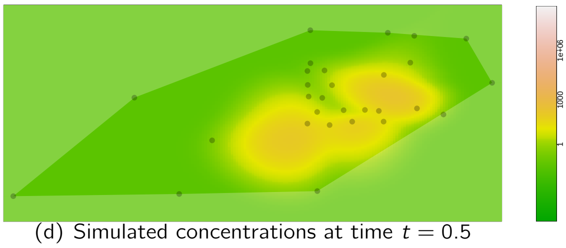

(d) Simulated concentrations at time $t = 0.5$

Figure S1: Flow model, initial concentrations and simulated concentrations for $t \in \{0.25, 0.5\}$ used in the simulation study. The simulated concentrations for $t = 0.7$ are shown in figure S2(a).

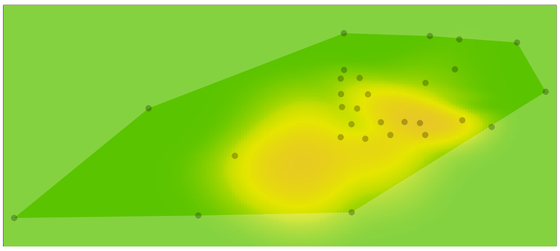

(a) Simulated true concentrations (base model at $t = 0.7$)

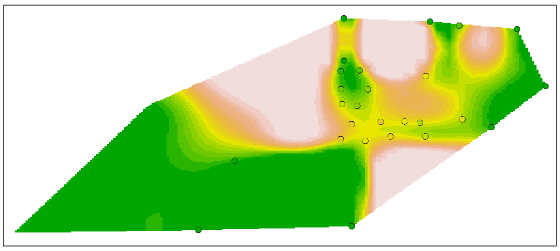

(b) aicc

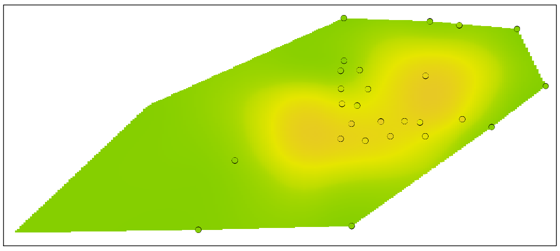

(c) bic

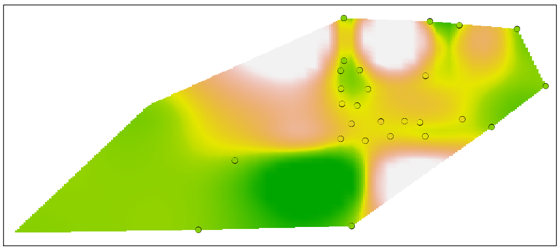

(d) gcv

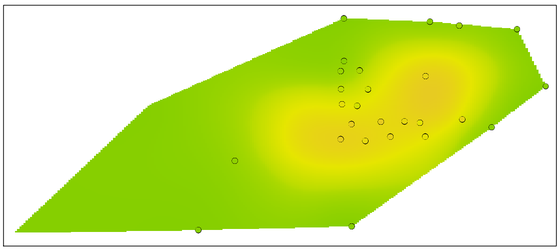

(e) Well-based cross-validation

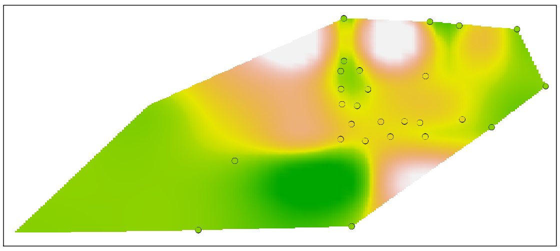

(f) Observation-based cross-validation

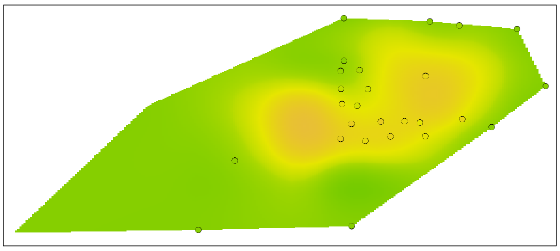

(g) Bayesian maximum-a-posteriori estimate

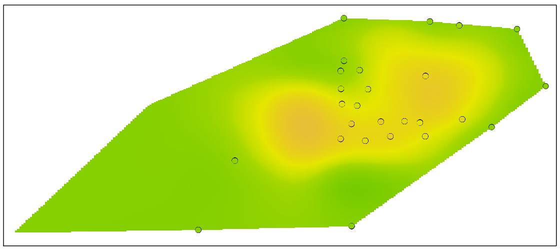

(h) Bayesian model averaging

Figure S2: Simulated true model (top left) as well as predictions obtained in one iteration of the simulation at time $t = 0.7$ using the wells from scenario 1. Each panel corresponds to the use of a different criterion for selecting the smoothing parameter.

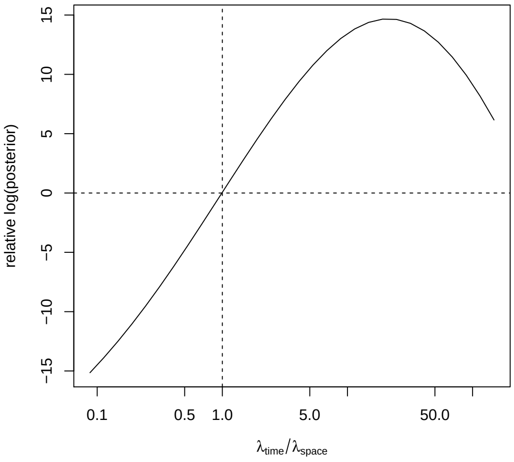


(a) Log-posterior (relative to log-posterior for equal smoothing parameters)

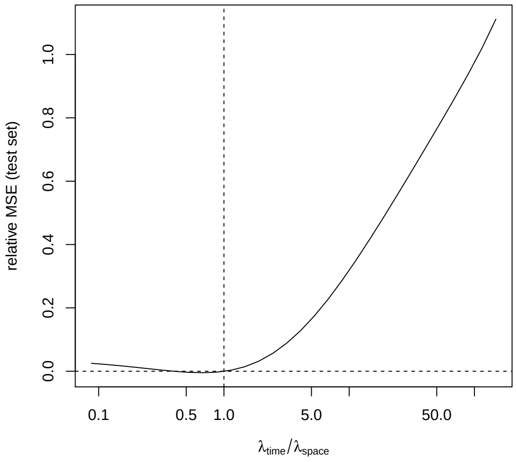


(b) Predictive MSE on the test set (relative to MSE for equal smoothing parameters)

Figure S3: Log-posterior and predictive MSE on the test set as a function of the ratio of smoothing parameters. Though the log-posterior has a mode for $\lambda$time $\approx 20 \cdot \lambda_{space}$, the predictive MSE on test data is minimal for $\lambda_{time} \approx \lambda_{space}$.

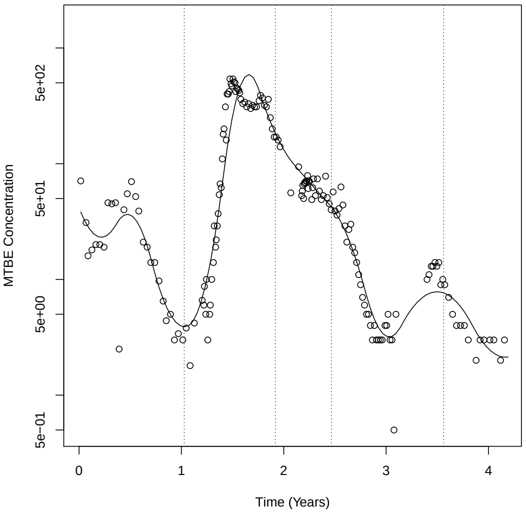


(a) Predicted concentration for well A

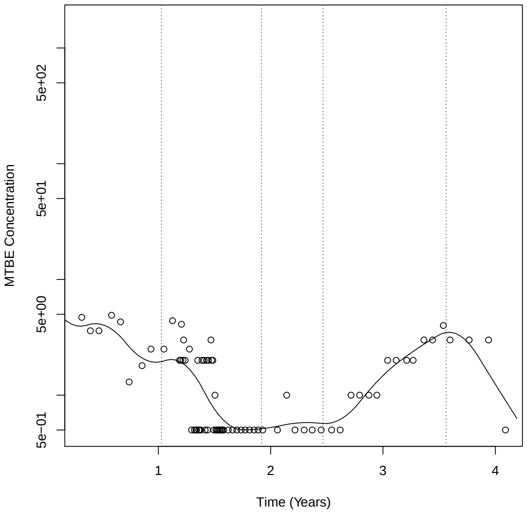


(b) Predicted concentration for well B

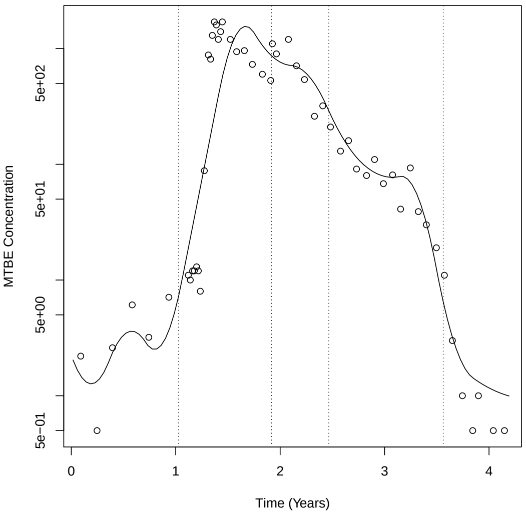


(c) Predicted concentration for well C

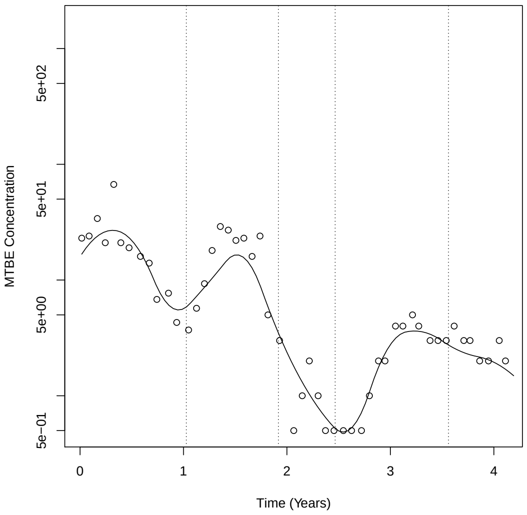


(d) Predicted concentration for well D

Figure S4: Predicted levels of mtbe concentration over time obtained using the map estimate of the smoothing parameter for four wells. The location of the wells is shown in Figure 4 (in the paper). The vertical dotted lines correspond to the time points used in Figure 5 (in the paper).

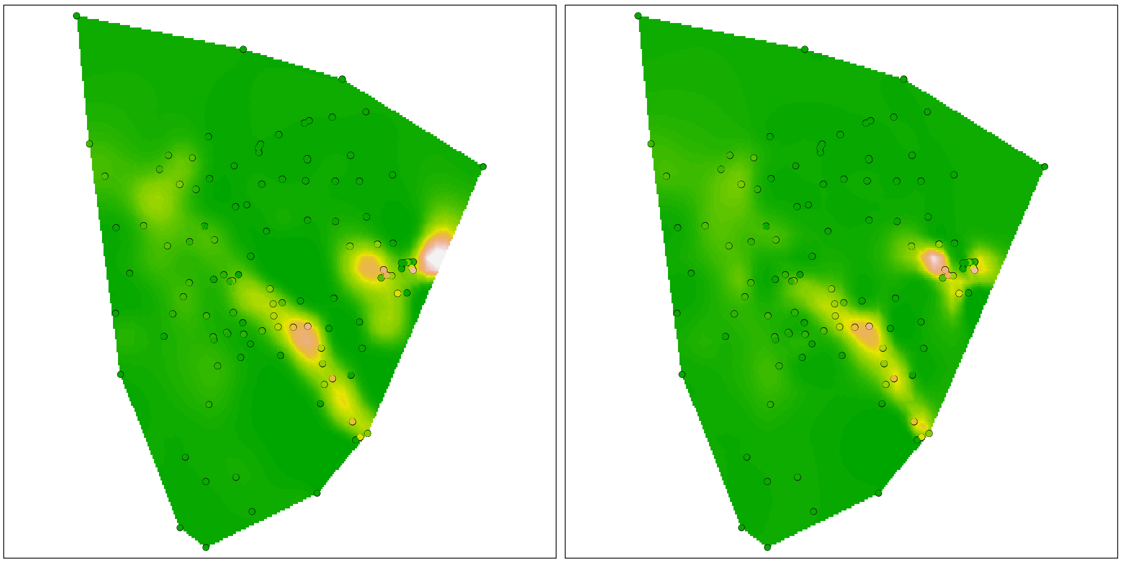

(a) Predicted concentration surface for day 1300 using a smaller number of basis functions

(b) Predicted concentration surface for day 1300 using a larger number of basis functions

Figure S5: Predicted levels of mtbe concentration across space for day 1300 obtained using a smaller number and a larger number of basis functions.
